# Proximal intestinal obstruction syndrome (PIOS) in a patient with cystic fibrosis: A case report

**DOI:** 10.1016/j.amsu.2020.11.063

**Published:** 2020-12-03

**Authors:** Carlos Antonio Morales Morales, Mauricio Gonzalez-Urquijo, Luis Fernando Morales Flores, Enrique Quevedo-Fernandez, Eduardo Alberto Guzmán Huerta, Martín Mauricio Virgilio Hernández-Torre

**Affiliations:** a Tecnologico de Monterrey, School of Medicine and Health Sciences, Dr. Ignacio Morones Prieto O 3000, Monterrey, 64710, Mexico; b Dr. Ignacio Morones Prieto O 3000, Monterrey, 64710, Mexico

**Keywords:** Cystic fibrosis, PIOS, DIOS, Case report, Bowel obstruction

## Abstract

**Introduction:**

Distal Intestinal Obstruction Syndrome is a rare complication in patients with cystic fibrosis, which characterized by the accumulation of viscid fecal material, combined with sticky mucous secretions located in the distal ileum adhere to the intestinal wall, causing complete bowel obstruction.

**Presentation of case:**

We report a case of a 45 years old patient with cystic fibrosis, who presented bowel obstruction secondary to accumulation of fecal material, combined with mucous secretions, in the mid-jejunum. A diagnostic laparoscopy was performed where a dilated jejunum was encountered with impaction of fecal content. Surgery was converted to open surgery, where a longitudinal enterotomy of 5 cm after the transition zone was created, evacuating manually the fecal material with mucous secretion. The patient evolved favorably, without complications.

**Discussion:**

We present a case of a patient with cystic fibrosis presenting with bowel obstruction due to a proximal intestinal obstruction syndrome, which can be diagnosed with the DIOS definition, with the only distinction of a more proximal location in the gastrointestinal tract, such as the stomach, the duodenum, or the jejunum.

**Conclusion:**

It is important for the clinician to know the existence of this syndrome at its different locations in the small bowel to treat accordingly.

## Highlights

• Proximal Intestinal Obstruction Syndrome is a rare complication in patients with cystic fibrosis.
• It is the accumulation of viscid feces with mucous secretions in the small bowel.
• It is important to know the existence of this syndrome to treat accordingly.

## 1 Introduction

Cystic fibrosis is a common, life-threatening, inherited disease that affects multiple organs, including the gastrointestinal tract, the reproductive and the respiratory system. It is an autosomal disease caused by mutation of the cystic fibrosis transmembrane conductance regulator (CFTR) protein, a cell membrane channel for the transport of chloride ions [1].

Distal Intestinal Obstruction Syndrome (DIOS) is a rare complication in CF patients characterized by the accumulation of viscid fecal material, combined with sticky mucous secretions located in the distal ileum, adhered to the intestinal wall [2,3]. The ESPGHAN CF Working Group defined DIOS as a complete bowel obstruction, evidenced by vomiting of bilious material, abdominal pain, and distention. On image studies air-fluid levels might be seen in the small intestine, corresponding to a bowel obstruction due to a fecal impaction in the ileo-caecum region [4]. DIOS has an incidence of 23.3–35.5 episodes per 1000 patients per year and a lifetime prevalence of 14–16% [5,6].

Only a few cases of DIOS have been reported in the literature. To our knowledge, no cases occurring in the proximal portion of the gastrointestinal tract have been reported before. The objective of this study is to report a case of a 45-year-old male patient with cystic fibrosis, who presented with intestinal obstruction in the jejunum due to impaction of viscid fecal material with sticky mucous secretion, treated in an academic hospital. A review of the literature is also performed. The work has been reported in line with the SCARE guidelines [7].

## 2 Case presentation

We present a 45-year-old male patient diagnosed with cystic fibrosis at three years of age, with no history of meconium ileus and no other relevant medical or surgical history. The patient's regular treatment included albuterol, multivitamins and pancreatic enzymes. He arrived at our emergency department due to dyspnea. Upon admission, an anteroposterior chest x-ray showed bronchiectasis and atelectasis due to chronic pneumopathy. A consolidation of the left inferior lobe and left pleural effusion was also seen, compatible with pneumonia. At that moment, heart rate was 82 beats per minute, respiratory rate 35 breaths per minute, and blood pressure 125/75 mmHg. Laboratory studies reported 12.4 × 10^3^ leukocytosis, hemoglobin 12 g/dl, platelets 223 × 10^3^/μl, C-reactive protein 15.28, glucose 117 mg/dl, and all other laboratories values were within normal parameters.

**Fig. 1:**
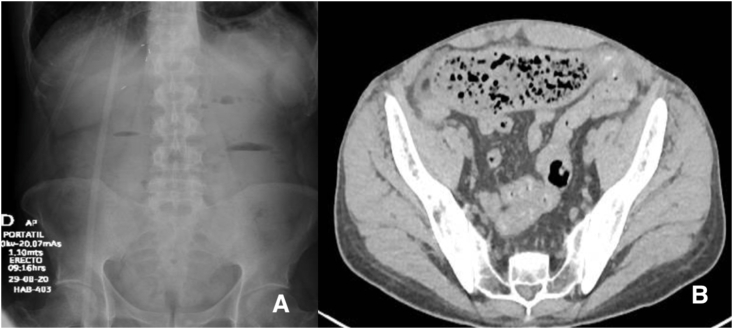
A) Abdominal x-ray. Air fluid levels in the small bowel, with no gas in the rectum. B) Abdominal computed tomography. Fecal content in the mid-jejunum.

The patient was started with antibiotics and supplement oxygen, and during his fourth day of hospitalization, the patient began with diffuse abdominal pain, bloating, nausea, vomiting of bilious material, and constipation. An abdominal x-ray revealed air fluid levels in the small bowel and no gas in the rectum **(**Fig. 1a). A computed tomography with oral contrast was taken, where dilatation of the stomach, the duodenum, and the proximal jejunum were seen, showing a transition zone in the mid-jejunum with no visible contrast past this segment, with fecal impaction at this site (Fig. 1b). DIOS was diagnosed, so polyethylene glycol was administered through the nasogastric tube. The nasogastric tube drainage was more than 1 L per day; therefore, on his third day due to failed medical treatment the patient was transferred to surgery.

**Fig. 2:**
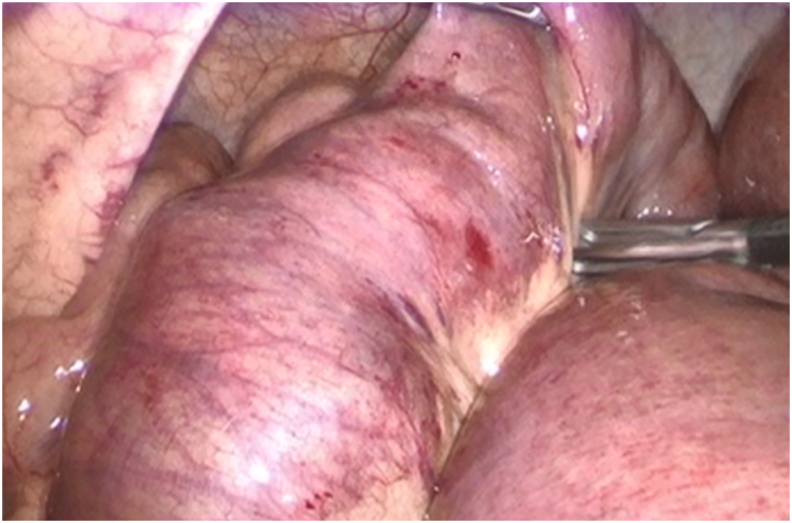
Laparoscopic view of fecal impaction at the mid-jejunum.

**Fig. 3:**
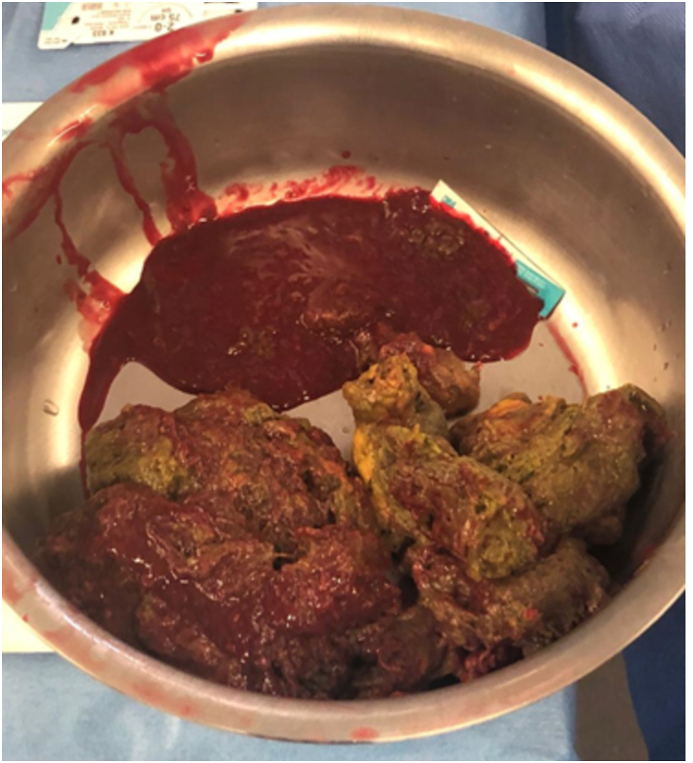
Manual fecal washout through enterotomy.

Upon acceptance from the patient, a diagnostic laparoscopy was done by the attending surgeon, running the small bowel from the ligament of Treitz to the ileocecal valve, observing a dilated mid-jejunum with impaction of abundant fecal content 100 cm from the ligament of Treitz (Fig. 2). The surgery was converted to laparotomy, performing a longitudinal enterotomy of 5 cm immediately after the fecal impaction manually extracting and washing the viscid fecal contents with sticky mucous secretions (Fig. 3). The enterotomy was repaired with a 3–0 PDS transversal suture. The patient evolved favorably, and the bowel obstruction was resolved. On post-op day two, he began oral intake and presented his first evacuation of semi-solid consistency. The patient was discharged from our service without further complications.

## 3 Discussion

DIOS is a well-described syndrome in patients with cystic fibrosis, characterized by the accumulation of viscid fecal material, combined with sticky mucous secretions located in the distal ileum, adhered to the intestinal wall, causing complete obstruction of the gastrointestinal lumen. This happens because the intestinal epithelium shares the same secretory processes seen in the airway of patients with cystic fibrosis, where chloride secretion, together with an inhibition of sodium absorption, acts as a driving force for abnormally fluid secretion [2].

Risk factors for presenting DIOS include a history of meconium ileus, poorly controlled fat malabsorption, dehydration, prolonged intestinal transit, and lung transplantation [8,9]. It typically presents with acute abdominal pain, abdominal distention, emesis, and a right lower quadrant mass on the physical exam might be palpated [4]. On image studies, abdominal X-rays are significant for fecal impaction, and computed tomography scans demonstrate small bowel dilatation with inspissated fecal material [10].

Differential diagnosis include severe constipation, appendicitis, and intussusception [11]. Medical treatment consists of osmotic laxatives such as polyethylene glycol or sodium meglumine diatrizoate (Gastrografin®), and adequate hydration [12]. Mucolytics, oral N-acetylcysteine, sodium docusate, and picosulphate may also be used [1]. Surgical intervention is generally accepted as a last resort, and laparotomy with washout via enterostomy should be tried before considering resection [13]. Cecostomy, right hemicolectomy, and small bowel resection, with primary anastomosis or diversion, have been reported in compromised bowels [14].

Alattar et al. [15] described a case with proximal intestinal obstruction in a cystic fibrosis patient in the mid-jejunum; however, it was noted to be due to kinking of the bowel causing vascular congestion. They speculate that the increased viscosity and prolonged intestinal transit time, characteristic of cystic fibrosis, resulted in inspissated fecal content in the proximal small bowel, which then acted as a lead point for obstruction. We reported a case of proximal intestinal obstruction in a cystic fibrosis patient fulfilling DIOS definition by ESPGHAN.

Since 1966 when the Cystic Fibrosis Foundation Patient Registry (CFFPR) was founded, clinicians have witnessed significant advances in the quality and quantity of life for patients living with cystic fibrosis [12]. We are facing long-lasting cystic fibrosis patients and new clinical presentations of syndromes, including this unique presentation of DIOS, in the proximal portion of the gastrointestinal tract.

In conclusion, small bowel obstruction in cystic fibrosis patients is often attributed to DIOS; however, whenever the obstruction occurs at the gastrointestinal tract's proximal portion, this syndrome should be rename as PIOS. Medical treatment should be the initial treatment, leaving surgical intervention whenever the conservative management fails, performing enterotomy and washout or intestinal resection if the bowel becomes compromised. It is important for the clinician to know the existence of this syndrome at its different locations in the small bowel to treat accordingly.

## Consent

Written informed consent was obtained from the patient for publication of this case report and accompanying images. A copy of the written consent is available for review by the Editor-in-Chief of this journal on request.

## Ethical approval

All procedures performed in this study were reviewed by Ethics Committee of Tecnologico de Monterrey under No. 22A and complied with the principles laid down in the Declaration of Helsinki.

## Sources of funding

This research received no external funding.

## Author contribution

Carlos Antonio Morales Morales—conception of the work, drafting of manuscript, final approval and agreement for the accountability of work. Luis Fernando Morales Flores—data interpretation, revising the manuscript, final approval and agreement for the accountability of work. Mauricio Gonzalez-Urquijo—data interpretation, revising the manuscript, final approval and agreement for the accountability of work. Eduardo Giasi Gonzalez, Eduardo Alberto Guzman Huerta and Martin Mauricio Virgilio Hernandez Torre—data interpretation, revising the manuscript, final approval and agreement for the accountability of work.

## Registration of research studies

Name of the registry: Proximal Intestinal Obstruction Syndrome (PIOS) in a Patient With Cystic Fibrosis: A New Syndrome
Unique Identifying number or registration ID: NCT04613063
Hyperlink to your specific registration (must be publicly accessible and will be checked): https://register.clinicaltrials.gov/prs/app/action/SelectProtocol?sid=S000ACKO&selectaction=Edit&uid=U0002XNC&ts=7&cx=-f901h8

## Guarantor

Mauricio Gonzalez Urquijo.

## Consent

Written informed consent was obtained from the patient for publication of this case series and accompanying images.

## Provenance and peer review

Not commissioned, externally peer-revieweds

## Declaration of competing interest

The authors declare no conflict of interest.
